# Single fluorescent protein-based Ca^2+ ^sensors with increased dynamic range

**DOI:** 10.1186/1472-6750-7-37

**Published:** 2007-06-29

**Authors:** Ekaterina A Souslova, Vsevolod V Belousov, John G Lock, Staffan Strömblad, Sergey Kasparov, Alexey P Bolshakov, Vsevolod G Pinelis, Yulii A Labas, Sergey Lukyanov, Lorenz M Mayr, Dmitriy M Chudakov

**Affiliations:** 1 Shemiakin-Ovchinnikov Institute of Bioorganic Chemistry, RAS, Miklukho-Maklaya 16/10, 117997 Moscow, Russia; 2 Karolinska Institutet, Department of Biosciences and Nutrition, Novum, SE-141 57 Huddinge, Sweden; 3 Department of Physiology School of Medical Sciences University of Bristol, BS8 1TD, Bristol, UK; 4 Scientific Centre for Children's Health RAMS, Lomonosovskii pr. 61/2, Moscow, Russia; 5 Bakh Institute of Biochemistry, RAS, Leninsky 33, 117071 Moscow, Russia; 6 Novartis Pharma AG, NIBR/DT/LDC, Lichtstrasse 35, CH-4002 Basel, Switzerland

## Abstract

**Background:**

Genetically encoded sensors developed on the basis of green fluorescent protein (GFP)-like proteins are becoming more and more popular instruments for monitoring cellular analytes and enzyme activities in living cells and transgenic organisms. In particular, a number of Ca^2+ ^sensors have been developed, either based on FRET (Fluorescence Resonance Energy Transfer) changes between two GFP-mutants or on the change in fluorescence intensity of a single circularly permuted fluorescent protein (cpFP).

**Results:**

Here we report significant progress on the development of the latter type of Ca^2+ ^sensors. Derived from the knowledge of previously reported cpFP-based sensors, we generated a set of cpFP-based indicators with different spectral properties and fluorescent responses to changes in Ca^2+ ^concentration. Two variants, named Case12 and Case16, were characterized by particular high brightness and superior dynamic range, up to 12-fold and 16.5-fold increase in green fluorescence between Ca^2+^-free and Ca^2+^-saturated forms. We demonstrated the high potential of these sensors on various examples, including monitoring of Ca^2+ ^response to a prolonged glutamate treatment in cortical neurons.

**Conclusion:**

We believe that expanded dynamic range, high brightness and relatively high pH-stability should make Case12 and Case16 popular research tools both in scientific studies and high throughput screening assays.

## Background

An increasing number of genetically encoded fluorescent sensors have recently been developed on the basis of GFP-like proteins [1-3]. However, currently available genetically encoded sensors are characterized by low signal intensity and limited dynamic range (maximum change in fluorescence ratio or intensity) [1,4,5], insufficient for routine applications in high throughput screening (HTS) assays and restricting sensitivity of precise single-cell studies. At the same time, genetically encoded sensors provide a much wider flexibility, allowing to be targeted to any chosen cellular compartment, to generate stable cell lines and transgenic animals, to be expressed in a particular tissue and/or in a temporally controlled manner under a specific promoter. Therefore, development of genetically encoded sensors characterized by increased dynamic range and signal intensity remains an actual task.

One of the most promising approaches to create genetically encoded sensors is based on the circularly permuted fluorescent protein (cpFP) fused to or inserted into sensitive domain(s) [6-12]. In the presence of an analyte or in response to a cellular event, sensitive domain(s) undergoe(s) structural rearrangements, inducing conformational changes of cpFP and resulting in its altered fluorescent properties. Circular permutation allows placing sensitive domains in a close proximity to the chromophore environment of cpFP within chimeric sensor construct. Therefore, conformational changes of the sensitive domains and their influence on the spectral properties of cpFP is direct and can lead to significant changes in the fluorescent signal.

In particular, such Ca^2+ ^sensors as GCaMPs [8,12] and Pericams [7] were constructed by fusing calmodulin and its target peptide M13 (fragment of myosin light chain kinase) to cpFP. In the presence of Ca^2+^, calmodulin binds to the M13 peptide, causing conformational changes in the vicinity of the chromophore and thereby influencing cpFP fluorescence. Similar sensors, named Camgaroos [6,9], are formally based on the non-permuted GFP, but contain an inserted calmodulin molecule at position Tyr145 of EYFP, which is essentially similar to the circular permutation approach.

In most cases, it was shown that spectral changes of the cpFP-based sensors fluorescence occur through the chromophore transition from the neutral (protonated) to the charged (anionic) form. Noteworthy, the same mechanism leads to 100–400 fold increase of green fluorescence after photoactivation of so called photoactivatable fluorescent proteins, PA-GFP [13] and PS-CFP [14]. This indicates that potentially the same dynamic ranges may be achieved for the cpFP-based fluorescent sensors, provided that the amino acid residues surrounding the chromophore are adapted properly and the conformational changes of the sensitive domains cause favorable conformational alterations in cpFP.

Here we describe the development of high dynamic range cpFP-based Ca^2+ ^sensors, that show up to 16.5-fold increase of the fluorescent signal (F/F_0_, fluorescence increase, fold) in response to Ca^2+^. These sensors are more pH stable compared to Flash-pericam [7] and GCaMP1.6 [8] and have approximately 3-fold higher dynamic range compared to GCaMPs [8,12]. We believe that the "fluorescent core" of the sensors reported herein may be employed to develop sensors of various specificity with increased dynamic range, allowing reliable quantitative analyses of cellular signaling pathways.

## Results and discussion

### 1. Analyzing the break point

**Figure 1:**
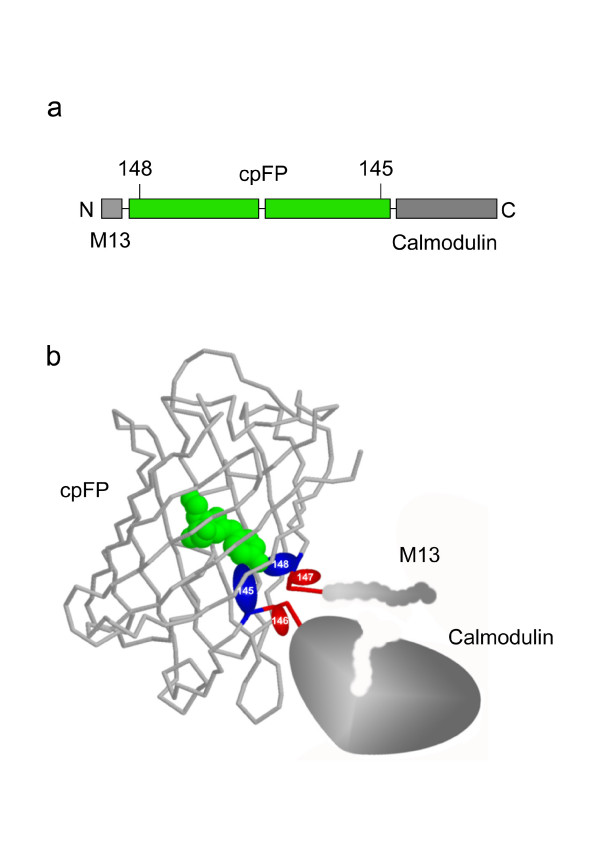
**Proposed model of a spatial organization of Pericams and GcaMPs**. a. Sensor constructs scheme. b. Proposed positional relationship of key amino acid residues (145–148) and sensitive domains (calmodulin and M13) within Ca^2+ ^sensors.

First we compared the break points of circular permutation within the β-barrel of fluorescent proteins used in Pericams and GCaMPs Ca^2+ ^sensors (Table 1A). Remarkably, amino acid residues corresponding to the positions 145 and 148 of the intact *Aequorea victoria *GFP (avGFP) (Figure 1a) were shown to substantially determine the fluorescent properties of the sensors [7,8]. Therefore, it is likely that in Pericams and GCaMPs the amino acid residues 148 and 145 are in a close proximity to the chromophore, similarly to the native avGFP (PDB ID: 1GFL) [15]. The amino acid residue preceding position 148 and the one following position 145 should be the outermost within the cpFP beta-barrel (Figure 1b). It can be presumed that the positional relationship of these key amino acid residues and the sensitive domains is common for the sensors described in Refs. [7,8,10-12] and this family of circularly permuted variants can be generally named cpFP147-146. A similar spatial organization can be proposed for the Camgaroo sensors [6,9].

**Table 1:** Key amino acid positions, linkers and *in vitro *Ca^2+ ^response of cpFP-based Ca^2+ ^sensors. Linker sites: linkers connecting M13 with cpFP and cpFP with calmodulin. cpFP is underlined, positions 148 and 145 are shown in bold. Data for Part A are from literature. See Methods section for the details concerning proteins purification and spectroscopy for Parts B and C

**Sensor**	**Linker sites**	**148**	**145**	**203**	**Maximum fluorescence response to Ca**^2+^**, fold, comments**
**Part A: previously developed sensors**					

**Pericam**	YNS**H**NVY---LEYN**G**TGDQ	H	G	Y	3,0 × increase
**Flash-periam**	YNS**H**NVY---LEYN**G**TGDQ	H	G	H	8,0 × increase low pH stability
**Ratiometric-pericam**	YNS**D**NVY---LEYN**G**TDQL	D	G	F	10,0 × ratiometric changes
**Inverse-pericam**	YNS**T**NVY---LEYN**G**TDQL	T	G	A	5,0 × decrease
**G18**	SSL**E**NVY---LEYN**G**TRDQ	E	G	T	4,3 × increase
**GCaMP1**	SSL**E**NVY---LEYN**T**RDQL	E	T	T	4,3 × increase brighter than G18 due to 145T
**GCaMP1.6**	SSL**E**NVY---LEYN**T**RDQL	E	T	T	4.9 × increase much brighter than GCaMP1
**GCaMP2**	SSL**E**NVY---LEYN**T**RDQL	E	T	T	5 × increase much brighter than GCaMP1.6

**Part B: our sensors developed as a result of 145/148 positions mutagenesis**					

**cps1**	SSL**N**NVY---LEYN**T**RDQL	N	T	F	Low increase
**cps1(A)**	SSL**N**NVY---LEYN**A**RDQL	N	A	F	1,4 × decrease
**cps1(S)**	SSL**N**NVY---LEYN**S**RDQL	N	S	F	1,5 × increase
**cps2**	SSL**E**NVY---LEYN**T**RDQL	E	T	F	14,5 × increase
**cps2(A)**	SSL**E**NVY---LEYN**A**RDQL	E	A	F	no response
**cps2(S)**	SSL**E**NVY---LEYN**S**RDQL	E	S	F	14,5 × increase
**cps4**	SSL**D**NVY---LEYN**T**RDQL	D	T	F	low increase
**cps4(S)**	SSL**D**NVY---LEYN**A**RDQL	D	A	F	7,2 × ratiometric changes
**cps4(A)**	SSL**D**NVY---LEYN**S**RDQL	D	S	F	8,0 × ratiometric changes

**Part C: Sensors developed after cpS2 random mutagenesis**					

**Case12**	SSL**E**NVY---LEYN**T**RDQL	E	T	F	12.0 × increase fast maturation
**Case16**	SSL**E**NVY---LEYN**S**RDQL	E	S	F	16.5 × increase

### 2. Adapting the chromophore environment within a Ca^2+ ^sensor

Looking for the sensor variants with expanded dynamic range, we generated a set of Ca^2+ ^sensor constructs which varied at positions 148 and 145. In GCaMPs and Pericams position 145 was either Gly or Thr (Table 1A). We presumed that two other amino acid residues with rather compact side chains, Ser and Ala, wouldn't cause spatial conflict at position 145, while they would alter the spectral properties of the sensor. Using site-directed mutagenesis, we ntroduced either Ser, Ala or Thr in position 145, in combination with Asp148 (which earlier resulted in Ratiometric Pericam), Glu148 (used in GCaMPs), or Asn148. The overall sensors design was similar to that of GCaMPs. We used M13 peptide, a calmodulin domain and linkers lengths identical to those reported for GCaMP1 [8], combined with cpFP described in Ref. [10] and originating from Ratiometric Pericam [7]. Importantly, all our sensor variants carried Phe at position 203, which was earlier shown to influence the fluorescent properties of cpFP-based indicators [7].

Our sensor variants and their responses to Ca^2+ ^are summarized in Table 1B. Indeed, the variation at positions 145 and 148 was shown to determine sensor characteristics to a large extent. Analogously to Ratiometric Pericam (carrying Gly145), combination of Asp148 and Phe203 resulted in dual-excitation spectrum (green fluorescence excited at 400 nm and 490 nm) and strong ratiometric response to Ca^2+ ^when coupled with Ala145 or Ser145. However, combination Asp148/Phe203/Thr145 resulted in poorly responding sensor.

At the same time, all variants carrying Asn148 or Glu148 showed a single 490 nm excitation peak. While Asn148-containing sensors demonstrated low dynamic ranges, combinations Glu148/Thr145 (cps2) and Glu148/Ser145 (cps2(S)) resulted in high dynamic range sensors with up to 14.5-fold increase of 490 nm excited green fluorescence in response to 1 mM Ca^2+^. Further, cps2 was optimized in respect of folding at 37°C using random mutagenesis approach, resulting in an enhanced variant with 12-fold fluorescence increase in response to Ca^2+^, named Case12. This variant was characterized by a fast maturation and high brightness and was chosen for further tests in living cells. Site directed mutagenesis T145S of Case12 resulted in a sensor named Case16, which was characterized by the highest dynamic range, with up to 16.5-fold fluorescence increase in response to Ca^2+ ^(Table 1C), though less effective maturation in *E. coli*.

### 3. Characterization of Case12 and Case16 *in vitro*

**Figure 2:**
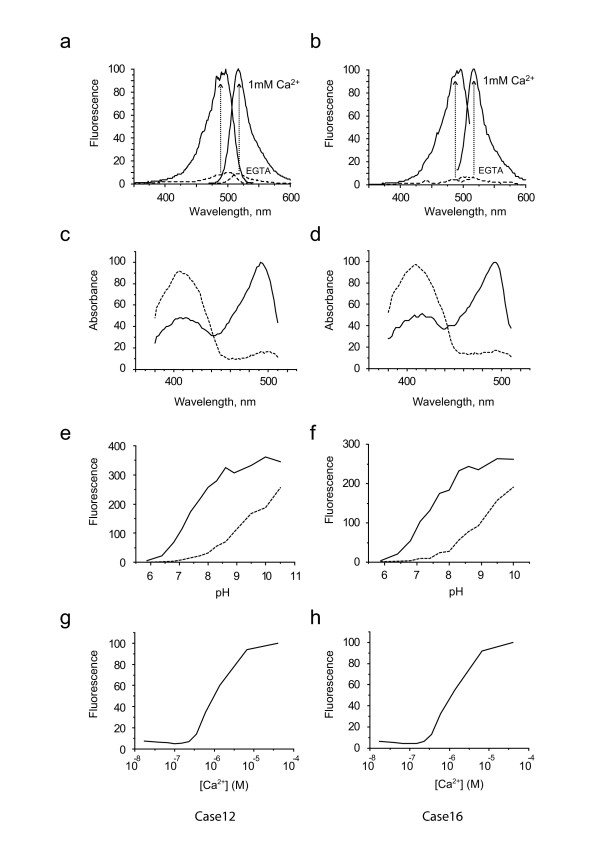
**Spectral characteristics of Case12 (a,c,e,g) and Case16 (b,d,f,h)**. a,b. Maximum fluorescent responses. Fluorescent spectra recorded in the presence of 0.5 mM EGTA (dashed lines) or 1 mM Ca^2+ ^(solid lines) at pH 7.4. c,d. Absorbance in the presence of 0.5 mM EGTA (dashed lines) or 0.4 mM Ca^2+ ^(solid lines) at pH 7.4. e,f. Dependence of sensors fluorescence on pH in the presence of 0.5 mM EGTA (dashed lines) or of 0.2 mM Ca^2+ ^(solid lines). g,h. Ca^2+ ^titration curves, at pH 7.4.

Spectral properties of Case12 and Case16 are summarized in Figure 2 and Table 2. Both sensors demonstrated record multi-fold increase of green fluorescent signal in response to 1 mM Ca^2+ ^(Figure 2a,b). Changes in the absorbance spectra in response to Ca^2+ ^(Figure 2c,d) were quite predictable from the observed fluorescence changes and reflected the redistribution between neutral and charged states of the chromophore in favor of the latter one. Similar responses, although with lower dynamic ranges, were reported earlier for Flash-pericam [7] and Camgaroo [6] sensors. Importantly, maximum brightness (in the presence of 1 mM Ca^2+^, at pH 7.4) reached 0.35 and 0.28 of that of EGFP for Case12 and Case16, respectively, which is essentially higher than that of Flash-pericam or GCaMP1.6 and comparable to GCaMP2 (Table 2).

**Table 2:** Spectral characteristics of GCaMP-like Ca^2+ ^sensors. Excitation/emission wavelengths, fluorescence quantum yield, extinction coefficient and pH stability in the presence of Ca^2+^are shown

Sensor	(F/F_0_)_max_	Excitation/emission wavelengths, nm	Quantum yield	Extinction coefficient, M^-1^cm^-1^	Relative brightness ^a^	pH stability (pKa)	K_d _for Ca^2+^, μM
Flash -pericam	8.0	494/514	0.20	16,900	0.10	7.9	0.7
GCaMP1	4.3	488/510	0.05	1,400	0.002	7.1	0.24
GCaMP1.6	4.9	488/509	0.79	3,800	0.09	8.2	0.16
GCaMP2	5.0	487/508	0.93	19,000	0.53	Nd	0.16
Case12	12.0	491/516	0.24	48,000	0.35	7.2	1.0
Case16	16.5	490/516	0.17	50,000	0.28	7.2	1.0

^a ^Calculated brightness compared to that of EGFP.

The common weak point of cpFP-based sensors is their low pH stability. For example, pKa (value of pH at which fluorescence brightness is 50% of maximum) for Pericams ranges from 7.9 to 8.5. Therefore, at physiological ranges of pH (7.2–7.5) such sensors exhibit lower brightness and dynamic range [7]. In contrast, the pKa values of Case12 and Case16 were shown to be 7.2 (in the presence of 0.2 mM Ca^2+^) close to that reported for GCaMP1 (Table 2 and Figure 2e,f). Therefore, Case12 and Case16 are characterized by notably higher pH-stability compared to GCaMP1.6 or Pericams (Table 2).

Further we measured Case12 and Case16 K_d _values for Ca^2+ ^binding at pH 7.4, using Molecular Probes assay (Calcium Calibration Buffer Kit #1). The apparent K_d _was found to be 1 μM for both sensors (Figure 2g,h), which lies within the physiological range of Ca^2+ ^concentrations. In most eukaryotic cells the free Ca^2+ ^concentration rests at approximately 100–200 nM and following activation of cellular signaling pathways cytosolic Ca^2+ ^levels are elevated up to several μM [16-19].

### 4. Characterization of Case12 and Case16 in living cells

**Figure 3:**
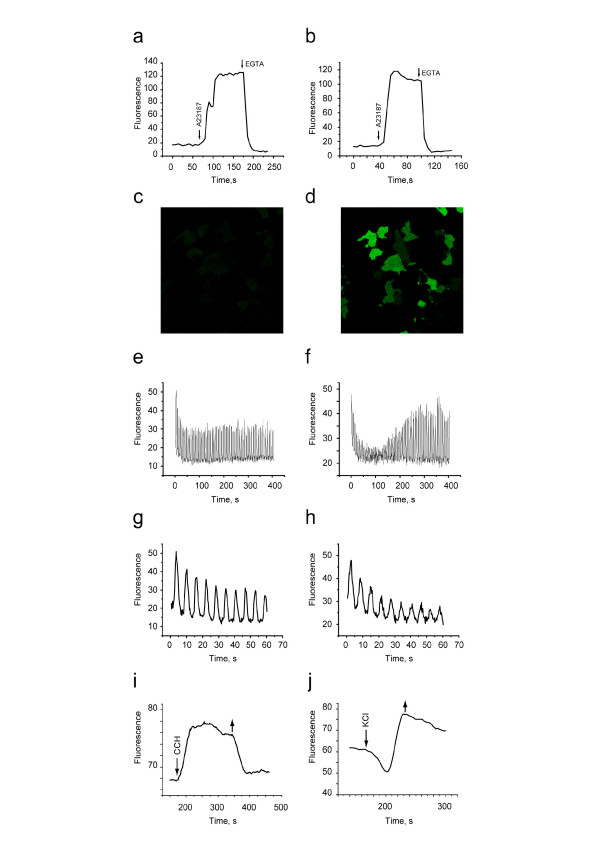
**Case12 and Case16 in living cells**. a,b. Typical responses of HeLa cells expressing Case12 (a) and Case16 (b) to Ca^2+ ^ionophore A23187. c,d. HeLa cells expressing Case12 are shown before (b) and after (c) ionophore addition. e-h. Fluorescence changes of M21 (human Melanoma-derived) cells expressing Case12 in response to 100 μM ATP. Images were captured every 0.294 sec on the confocal microscope. e,f. Individual responses of two selected cells within 400 s after ATP addition. g,h. The same cells, first 60 s of response. i. PC12 cells response to 500 uM carbachol (CCH). j. PC12 cells response to 30 mM KCl. For i and j first and second arrows indicate the moments of a compound addition and of washing start, respectively.

First, we checked maximum fluorescence response for both sensors in living HeLa cells. For this purpose the sensors were cloned into the N-vector (Evrogen), driving gene expression in eukaryotic cells. Cells transfected with Case12 sensor variant demonstrated relatively weak green fluorescence, which could be detected under a FITC filter set or upon excitation with a 488 nm laser line, emission collected at 500–540 nm (Leica microscope DM IRE2, confocal TCS-SP2, objective HCX-PL-APO-63x/1.40-0.60/OIL). Addition of 30 μM Ca^2+ ^ionophore A23187, allowing Ca^2+ ^(2 mM Ca^2+ ^in the medium) to enter cells, typically resulted in 5–6-fold increase in green fluorescence brightness. Further addition of 20 mM EGTA removed Ca^2+ ^and decreased the fluorescence signal close to baseline level, with the final contrast of 11–12-fold. Very similar results with 7–9 fold increase in the presence of ionophore and the final contrast of 13–15 fold were obtained for Case16 (Figure 3a–d). In analogues experiments with GCaMP2, one of the best reported cpFP-based Ca^2+ ^sensors [12] (a kind gift from Prof. Michael I. Kotlikoff and Dr. Junichi Nakai), the corresponding contrasts were 2.5-fold increase in the presence of ionophore and 5-fold decrease after addition of EGTA, corresponding with the reported dynamic range [12].

Further we tested the response of the sensors to the less pronounced Ca^2+ ^changes under physiological conditions. M21 human melanoma cells expressing Case12 displayed a nice high dynamic range response to ATP at a final concentration of 100 μM (Figure 3e–h). This experiment clearly showed that Case12 fluorescence response to Ca^2+ ^oscillations is fast and reversible. It also demonstrated that the sensor responds to changes in Ca^2+ ^concentration in living cells in the nanomolar range.

In another experimental set-up, we monitored Ca^2+ ^responses in PC12 cells. Transfected cells were grown at 37°C and NGF (nerve growth factor) was added 24 hours before the experiment in order to induce differentiation. In the course of imaging, cells were perfused with carbogen-saturated ACSF (artificial cerebro-spinal fluid – modified Ringer) [20] within a 34°C chamber. Figure 3i shows the Ca^2+ ^response of Case12 sensor to carbachol (at concentration 500 μM, between 170 and 350 s). After 10 min of wash, 30 mM KCl was added to the same cells (Figure 3j).

Further we compared Ca^2+ ^responses of Case12, Case16 and a well-known chemical probe for Ca^2+^, Oregon Green 488 BAPTA-1 (Molecular Probes; loaded at a final concentration of 7 μM for 4 hours) to 30 mM KCl in PC12 cells. Initial fluorescence brightness of the genetically encoded sensors and Oregon Green were approximately at the same level, and amplitudes of responses to Ca^2+ ^were very similar. This indicated that Case12 and Case16 are comparable with chemical probes for Ca^2+ ^in respect of signal brightness and dynamic range.

**Figure 4:**
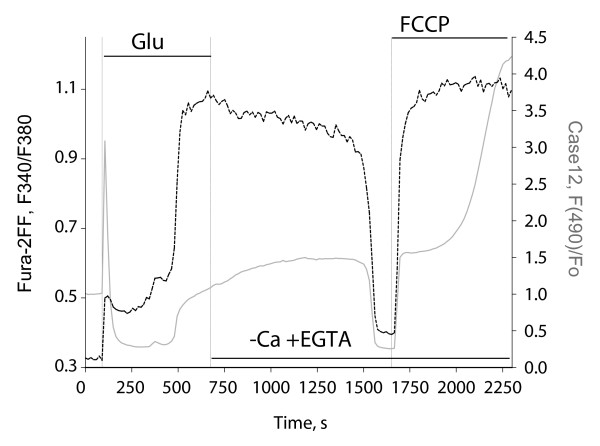
**Ca^2+ ^response to a prolonged glutamate treatment in cortical neurons**. Fluorescent signals of Case12 (gray solid line, excitation at 490 nm, 515–565 nm emission filter) and fura-2FF (black dashed line, ratio of 340 nm and 380 nm excited green fluorescence, 505–530 nm emission filter) are shown.

To further evaluate the sensors in comparison with chemical probes, we applied Case12 for measuring the Ca^2+ ^response to a prolonged glutamate treatment (100 μM glutamate, 10 μM glycine) in cortical neurons. Figure 4 illustrates typical response of cortical neuron transfected with Case12 and loaded with fura-2FF, which is a widely used chemical Ca^2+ ^indicator. Clear spectral differences allowed monitoring of fluorescence of both Case12 and fura-2FF sensors independently, in sequential excitation mode. While fura-2FF response was tracked by 340 nm and 380 nm excited green fluorescence brightness (505–530 nm emission filter), Case12 fluorescence was monitored using 515–565 nm emission filter, with excitation at 490 nm. Signals were measured within the same cells in parallel (Figure 4). Stimulation of glutamate receptors induced primary increase in the cytosolic Ca^2+ ^concentration ([Ca^2+^]_c_) that after a certain delay was superseded by the secondary [Ca^2+^]_c _elevation referred to as loss of Ca^2+ ^homeostasis [21,22]. The signal of Case12 clearly detected both phases of glutamate-induced [Ca^2+^]_c _elevation (Figure 4). It is fair to note that the latent period between these phases was seen as a pronounced decrease in the Case12 fluorescence, while the fura-2FF ratio showed increased [Ca^2+^]_c _level during this period. Taking into account that cpFP-based sensors are sensitive to pH changes, we presume that this decrease in Case12 signal is caused by glutamate-induced decrease in cytosolic pH [21,23,24]. Glutamate washout by Ca^2+^-free EGTA (100 μM)-containing solution induced delayed [Ca^2+^]_c _recovery. Consequent application of protonophore FCCP (1 μM) in Ca^2+^-free medium caused mitochondrial depolarization and released Ca^2+ ^accumulated by mitochondria during glutamate treatment [25,26]. Both processes were clearly monitored by the Case12 signal (Figure 4). In spite of the dramatic pH changes during glutamate treatment of cortical neurons, Case12 clearly monitored all the changes in ([Ca^2+^]_c_).

## Conclusion

Deeper understanding of cpFP-based sensors mechanics would contribute significantly to the development of "ideal" cpFP-based sensors, characterized with high brightness, dynamic range, pH- and photo-stability. We believe that proposed spatial arrangement of cpFP-based sensors (Figure 1B) is close to the reality and that this model will be helpful for the further development of cpFP-based sensors for various analytes and cellular signaling events. However, solution of the crystal structure for one of the cpFP-based sensors remains an actual task in this respect.

Developed high dynamic range sensors Case12 and Case16 set a new record among cpFP-based and, in general, among any GFP-based sensors reported up to date [1]. The most contrasting of the GFP-based FRET-indicators, Ca^2+ ^sensor YC3.60 [27], reaches a 6.6-fold ratiometric fluorescence changes. The most contrasting of the reported cpFP-based sensors, Flash-pericam, reaches a 8-fold increase of fluorescence [7], whilst yielding to Case12 and Case16 in respect of pH-stability and signal brightness (Table 2). The combined advantages should provide reliable monitoring of Ca^2+ ^signaling and suggest that Case12 and Case16 will become very popular research tools both in scientific studies and HTS assays and might be able to replace some of the currently used chemical Ca^2+ ^probes.

## Methods

### Cloning and gene construction

Sensor constructs generation and site directed mutagenesis were performed by overlap-extension PCR [28], with primers containing the appropriate target substitutions. The cDNA coding the circularly permuted green fluorescent protein was amplified from the HyPer sensor [10]. The sequence of the M13 peptide (from the chicken smooth muscle myosine light chain kinase) was generated by step-out PCR. The sequence of calmodulin was amplified from a human cDNA library. Clontech Diversity PCR Random Mutagenesis kit was used for random mutagenesis, in conditions optimal for 7 mutations per 1000 bp. For bacterial expression, a PCR-amplified *BamHI/HindIII *fragment encoding sensor variant was cloned into the pQE30 vector (Qiagen). For expression in eukaryotic cells, PCR-amplified *AgeI/NotI *fragment encoding the corresponding sensor variant was swapped for TurboGFP within the pTurboGFP-N vector (Evrogen).

### Protein expression and *in vitro *spectroscopy

Proteins fused to the N-terminal polyhistidine tag were expressed in *E. coli *XL1 Blue strain (Invitrogen). The bacterial cultures were centrifuged and the cell pellets re-suspended in 20 mM Tris-HCl, 100 mM NaCl, pH 7.4 buffer and lysed by sonication. The recombinant proteins were purified using TALON metal-affinity resin (Clontech) followed by a desalting step over gel-filtration columns (Bio-Rad). Absorption spectra were recorded with a Beckman DU520 UV/VIS Spectrophotometer. A Varian Cary Eclipse Fluorescence Spectrophotometer was used for measuring excitation-emission spectra. For calculation of the molar extinction coefficients, we relied on the determination of the mature chromophore concentration. Proteins were alkali-denatured with an equal volume of 2 M NaOH. Under these conditions, the GFP chromophore absorbs at 446 nm and its molar extinction coefficient equals 44,000 M^-1^cm^-1^. Absorption spectra for native and alkali-denatured proteins were analyzed. Based on the absorption of denatured proteins, molar extinction coefficients for the native states were estimated. For determination of the quantum yield, the fluorescence of the mutants was compared with equally absorbing EGFP (quantum yield 0.60 [29]). Ca^2+ ^titrations were performed using buffers identical to Calcium Calibration Buffer Kit #1 (Molecular Probes), yielding the free Ca^2+ ^concentrations from zero to 39 μM. Fluorescence emission spectra (excited at 490 nm) were recorded at 22°C. pH titrations were performed by using a series of buffers. For each pH value an aliquot of purified protein was diluted in an equal volume of the corresponding buffer solution either in the presence of 5 mM EGTA or 200 μM CaCl_2_, and the fluorescence brightness was measured (fluorescence excited at 490 nm and detected at 520 nm).

### ATP-response

M21 human melanoma cells were transfected with FuGENE reagent (Roche), grown for 48 h in RPMI medium in the presence of 10% FBS (Invitrogen) in a CO_2 _incubator at 37°C, 5% CO2, and then incubated for 1 h before imaging in 10% FBS Ham's F-12 medium (Invitrogen). Imaging was then performed in Ham' s F-12 medium by confocal laser scanning microscopy (LSM510, Carl Zeiss Microimaging, Inc.) ATP was added to a final concentration of 100 μM and the fluorescence of selected transfected cells was then monitored every 0.294 sec. Data were analyzed using ImageJ software (NIH, Bethesda).

### Primary cultures of cortical neurons

Cortical neurons were prepared from 1–3 days old newborn Wistar rats. Cortical tissue was minced in ice-cold Krebs salt solution. (mM: 130 NaCl, 5.4 KCl, 20 HEPES, 0.4 KH_2_PO_4_, 15 Glucose, 0.5 MgSO_4 _and 3 mg/ml BSA (Sigma), pH 7.4), then the tissue was digested in Krebs solution with 0.8 mg/ml trypsin 1–300 (ICN) for 15 min at 36°C. Trypsin was inactivated by washing with Krebs solution containing 0.08 mg/ml trypsin inhibitor (Sigma) and 0.01 mg/ml DNase (Roche). Cells were dissociated by trituration and pelleted in Krebs solution containing 0.5 mg/ml trypsin inhibitor and 0.08 mg/ml DNase. Cells were then resuspended in Neurobasal Medium (Gibco) with supplement B-27 (Gibco), GlutaMax (Gibco) and penicilline/streptomycine (Gibco) and plated onto 25 mm glass coverslips, coated with poly-D-lysine (Sigma). After 5–7 days in the primary culture, cells were transfected with Case12 using Lipofectamine 2000 (Invitrogen, Carlsbad, CA), following the protocol recommended by the manufacturer. Fluorescence analyses were carried out 2 d after transfection.

### Imaging of fura-2FF and Case12 signals

The neurons transfected with Case12 were loaded for 40 min with 3 μM fura-2FF/AM (Teflabs, Austin, TX, USA) in the incubator in the presence of cell culture medium. Images were acquired on an epifluorescence inverted microscope Axiovert 200 (Zeiss, Germany) equipped with a 20× fluorite objective. [Ca^2+^]_c _was monitored in single cells using excitation light provided by a Xenon arc lamp, the beam passing sequentially through 10 nm bandpass filters centered at 340, 380 and 490 nm housed in a computer-controlled filter wheel (Sutter Instrument Co., CA, USA). Emitted fluorescence light was reflected through a 505–530 nm filter (fura-2FF) and 515–565 nm filter (Case12) placed in computer-controlled filter wheel. Images were acquired by CCD camera (Roper Scientific, USA). All imaging data were collected and analyzed using the Metafluor 6.1 software (Universal Imaging Corp., USA). The fura-2FF data are presented as the ratio of light excited at 340 nm/380 nm.

### Experimental procedures with primary cultures of cortical neurons

The coverslips with cell culture were placed into the 300 μl experimental chamber at room temperature (25°C) and washed with a standard physiological recording saline containing (mM): 140 NaCl, 5.4 KCl, 2 MgCl_2_, 2 CaCl_2_, 5 glucose and 20 HEPES, pH adjusted to 7.4 with NaOH. The solution in the chamber was removed by a peristaltic pump. The solutions were added into the chamber with the aid of a pipette. Washout of solutions was made three times to completely remove old solution from the chamber.

## Abbreviations

GFP, green fluorescent protein; avGFP, *Aequorea victoria *green fluorescent protein, EGFP, enhanced green fluorescent protein; cpFP, circularly permuted fluorescent protein; FRET, fluorescence resonance energy transfer, NGF, nerve growth factor; ACSF, artificial cerebro-spinal fluid.

## Authors' contributions

EAS carried out the molecular genetic work, protein purification and microscopy studies. VVB carried out part of the molecular genetic work. JGL and SS took part in the microscopy studies on living cells and drafted the manuscript. SK carried out part of the microscopy studies on living cells. APB and VGP carried out experiments with primary cultures of cortical neurons and partially drafted the manuscript. YAL, SL and LMM participated in the design of the study and drafted the manuscript. DMC conceived of the study, participated in its design and coordination, wrote the manuscript, and carried out part of the microscopy studies on living cells. All authors read and approved the final manuscript.
